# Sharing perspectives on feedback: a combined resident-faculty workshop

**DOI:** 10.1186/s12909-022-03253-6

**Published:** 2022-03-22

**Authors:** Bo Kim, Aishwarya Rajagopalan, Edward M. Tabasky, Sparsha S. Reddy, David R. Topor

**Affiliations:** 1 VA Boston Healthcare System, 150 South Huntington Ave., Boston, MA 02130 USA; 2 Harvard Medical School, 25 Shattuck St., Boston, MA 02115 USA; 3 ICL East New York Health Hub, 2581 Atlantic Ave., Brooklyn, NY 11207 USA; 4 VA Bedford Healthcare System, 200 Springs Rd., Bedford, MA 01730 USA

**Keywords:** Residency, Feedback, Faculty development, Professionalism, Lifelong learning

## Abstract

**Background:**

Feedback is essential to medical education. Although the need for effective feedback delivery is well known, more recent focus is on understanding and strengthening the faculty-trainee relationship within which the feedback process is carried out. The authors developed and implemented a combined resident-faculty feedback workshop within a psychiatry residency training program to enhance participants’ understanding of challenges residents and faculty experience with the feedback process.

**Methods:**

The one-hour workshop consisted of small group activities and large group discussions, focused on (i) feedback challenges for both residents and faculty and (ii) potential ways to address identified challenges. Participants completed pre-and post-workshop questionnaires to rate their level of understanding of, and answer open-ended questions regarding, feedback challenges. Mixed-methods assessment of questionnaire responses examined quantitative rating changes from pre- to post-workshop, as well as emergent qualitative themes from the open-ended responses.

**Results:**

From a pool of 30 workshop participants, 26 completed each of the pre- and post-workshop questionnaires. Overall, participants were satisfied with the programming. Important considerations for the feedback process were (i) specific/constructive/timely feedback, (ii) meeting logistical/administrative feedback requirements, (iii) setting norms/expectations of effective/routine feedback, and (iv) relational/emotional considerations surrounding feedback. It appeared both faculty and residents were able to increase perspective taking about how the other group perceived the feedback process.

**Conclusions:**

This pilot project is one of the first to examine a joint resident-faculty workshop focused on understanding how faculty and residents can interact to better understand each other’s perspective on the feedback process. Further work in this area is needed to identify common misperceptions and design programming to help correct them. Further research is also needed to examine the impact of such programming on the feedback process.

## Background

Feedback is an essential component of medical education [1] and is a growing focus of both medical training and faculty development programs [2, 3]. A 2017 scoping literature review on trainee feedback in medical education (of 650 peer-reviewed articles published from 1980 through 2015) found that over 95% of the reviewed articles were on methods for giving feedback [4]. This finding reflects the field’s (i) awareness of challenges faculty face providing effective feedback to trainees and (ii) desire for guidance on how to provide effective feedback.

Although the need for effective strategies to deliver feedback is well known, more recent focus is on understanding and strengthening the faculty-trainee relationship within which the feedback is given [5]. Specifically, feedback is increasingly viewed as a complex interaction between the faculty member and the trainee, and its effectiveness is considered to be impacted by a multitude of factors surrounding the two entities [6, 7] – e.g., (i) expectations about what constitutes feedback, (ii) anxiety about potential relational consequences of feedback, and (iii) practical challenges of incorporating feedback procedures into demanding training and professional schedules.

Importantly, there are common discrepancies in how faculty and trainees perceive feedback [8, 9]. This lack of a shared understanding may contribute to defensiveness and anger that threaten feedback effectiveness [5, 10–12]. Recent studies have focused specifically on understanding medical resident and/or faculty perceptions of feedback [5, 13, 14], through qualitative analyses of data from interviews or focus groups. As the next step, the field needs to identify effective programming to help faculty and residents understand and appreciate others’ perspectives on feedback and develop a shared model of the feedback process.

To address this gap, the authors developed and implemented a combined resident-faculty feedback workshop within an Accreditation Council for Graduate Medical Education (ACGME)-accredited psychiatry residency training program. The main goal of the workshop was to enhance participants’ understanding of both unique and similar challenges residents and faculty experience with feedback. Guided by participant-centered learning concepts, the authors designed the workshop to be interactive and discussion-driven. We are not aware of similar workshops that combined both faculty and residents to discuss feedback. Here we describe the components of this workshop and its impact on participants.

## Methods

The combined resident-faculty feedback workshop was a part of ongoing local quality improvement efforts at the Harvard South Shore Psychiatry Residency Training Program (HSS), located at the VA Boston Healthcare System (VABHS), to better characterize and improve the training program’s feedback practices. As part of these previous efforts, both residents and faculty expressed interest in a combined resident-faculty workshop to gain a better understanding of how each group perceives the process of giving and receiving feedback. VABHS has determined that HSS feedback improvement efforts are a quality improvement activity that is classified as non-research, requiring no written consent, further oversight, or review.

### Workshop content

All HSS residents and faculty were invited to participate in the hour-long workshop. Participants were given six months advance notice of the workshop, to prioritize attendance. The workshop was facilitated by authors BK, AR, and DT, and consisted of the following components – (i) pre-workshop questionnaire, (ii) workshop agenda, (iii) feedback challenges for both residents and faculty (small group activity followed by large group discussion), (iv) potential ways to address identified challenges (small group activity followed by large group discussion), and (v) post-workshop questionnaire.

#### Pre-workshop questionnaire

Participants completed a paper-based questionnaire prior to the workshop. Participants were asked to indicate their role in the training program (postgraduate year or faculty), and to rate their level of agreement (Strongly Disagree, Disagree, Neutral, Agree, or Strongly Agree) with the following rating-based (RB) items:

- RB1. I understand challenges that residents face in receiving feedback from faculty.
- RB2. I understand challenges that faculty face in providing feedback to residents.
- RB3. I feel comfortable talking with both faculty members and residents about feedback.
- RB4. I am able to identify ways to address challenges related to giving and receiving feedback.

The questionnaire also included two open-ended (OE) questions:

- OE1. What is most challenging for residents in receiving feedback from faculty?
- OE2. What is most challenging for faculty in providing feedback to residents?

#### Workshop agenda

Participants formed groups of three to five people to engage in the workshop activities. Each group consisted of at least one faculty member and a mix of residents from different postgraduate years. Workshop facilitators stressed the brainstorming (rather than decision-making) nature of the activities (e.g., by requesting that participants consciously refrain from responding with “but” or “however” to a fellow group member) to help ensure both residents and faculty were heard within the small groups.

#### Activity 1 of 2: Feedback challenges for both residents and faculty

Within the small groups, participants were asked to discuss the following questions, in this specific order:

- What do residents think is most challenging for faculty providing feedback?
- What do faculty think is most challenging for residents receiving feedback?
- What do residents feel is actually most challenging in receiving feedback?
- What do faculty feel is actually most challenging in providing feedback?

After approximately 10–15 min, participants reconvened as a large group. Facilitators led an open discussion about these questions, asking representatives from each of the small groups to identify key findings. Adhering to best practices for participant-centered learning, the facilitators recorded the shared topics on a PowerPoint slide that was shown in real-time to allow participants to ensure their thoughts were accurately captured.

#### Activity 2 of 2: Potential ways to address identified challenges

Within their original small groups, participants were asked to brainstorm potential ways to address both resident and faculty challenges identified in Activity 1. Small groups were asked to allow each member to offer their thoughts, and were reminded of the brainstorming nature of the activity (as mentioned above under the “Workshop agenda” subsection). Similar to Activity 1, after approximately 10–15 min, participants reconvened as a large group, and the facilitators led an open discussion of brainstormed ideas while recording the ideas on a projected slide.

#### Post-workshop questionnaire

Participants were asked to complete a paper-based post-workshop questionnaire. The post-workshop questionnaire contained the same questions as the pre-workshop version. Participants were also asked to rate their level of agreement (Strongly Disagree, Disagree, Neutral, Agree, or Strongly Agree) with the following rating-based (RB) items:

- RB5. Overall, I was satisfied with this workshop.
- RB6. I learned new knowledge and skills from this workshop.
- RB7. I will be able to apply the knowledge and skills learned to improve my job performance.
- RB8. The scope of the workshop was appropriate for my professional needs.
- RB9. I would recommend this workshop to others.
- RB10. The learning activities and/or materials were effective in helping me learn the content.
- RB11. The content was presented in a fair and unbiased manner.

The questionnaire included three additional open-ended (OE) questions:

- OE3. What did you find to be most useful from today’s workshop and why?
- OE4. What would have made today’s workshop more useful?
- OE5. What would you like to see future feedback enhancement workshops focus on?

### Participation by residents and faculty

Thirty participants attended the workshop. This included 21 residents (70%) and 9 faculty (30%). Residents were evenly distributed across postgraduate years. Adhering to the quality improvement designation of this work, the authors did not (i) prevent participants from joining the workshop late or leaving early from the workshop, which was the case for a few participants, or (ii) collect demographic data from participants that would not be directly used for conducting the workshop.

### Analysis of questionnaire responses

The authors calculated the proportion of respondents selecting the Strongly Agree or Agree rating for each RB item. Changes in these proportions from pre-workshop to post-workshop were examined. Aligning the five-category rating (i.e., from Strongly Disagree to Strongly Agree) to a five-point Likert scale (from one to five, respectively), the authors calculated mean Likert scale responses and standard deviations. Two-sample t-tests were used to compare mean Likert scale responses between the pre- and post-workshop questionnaires.

A qualitative thematic analysis was conducted on responses to the OE questions. This analysis was conducted based on Guest et al.’s four steps in undertaking thematic analysis [15], as outlined by Chapman et al. for applications to healthcare research [16] – (i) getting acquainted with data, (ii) recognizing emergent themes, (iii) subdividing/combining and grouping themes into categories, and (iv) conceptualizing the model that interrelates the themes. For OE questions on both the pre- and post-workshop questionnaires, changes in their associated emergent themes were also assessed.

This mixed quantitative–qualitative approach followed a sequential complementary connection of the quantitative and qualitative components of data collection and analysis [17]. In other words, (i) the data were simultaneously collected and analyzed, and (ii) quantitative and qualitative data were complementarily used to provide depth and breadth of understanding, respectively, where (iii) the qualitative data from the open-ended questions built on the quantitative data from the rating-based items.

## Results

Twenty-six respondents, including 18 residents, 7 faculty, and 1 respondent who did not specify their role, completed the pre-workshop questionnaire. Twenty-six respondents, including 16 residents, 6 faculty, and 4 respondents who did not specify their role, completed the post-workshop questionnaire.

### Rating-based questionnaire responses

Results of the RB questionnaire items that were a part of both the pre- and post-workshop questionnaires are displayed in Table 1. For each of Items RB1 through RB4, a greater proportion of respondents selected “Strongly Agree” or “Agree” on the post-workshop questionnaire than on the pre-workshop questionnaire. The mean Likert scale responses also increased for each of these four items from pre- to post-workshop. The increases for overall responses and for resident responses were statistically significant.

**Table 1:** Results from rating-based pre- and post-workshop questionnaire items

	**Pre-workshop questionnaire** **(*n* = 26 overall, including 18 residents, 7 faculty, and 1 unspecified)**						**Post-workshop questionnaire** **(*n* = 26 overall, including 16 residents, 6 faculty, and 4 unspecified)**						**Change from pre- to post-workshop**					
**Rating-based (RB) questionnaire item**	Proportion of n selecting the Strongly Agree or Agree rating			Mean (SD) Likert scale responses^a^			Proportion of n selecting the Strongly Agree or Agree rating			Mean (SD) Likert scale responses^a^			Proportion of n selecting the Strongly Agree or Agree rating			Mean (SD) Likert scale responses^a^		
	**Overall**	Residents	Faculty	**Overall**	Residents	Faculty	**Overall**	Residents	Faculty	**Overall**	Residents	Faculty	**Overall**	Residents	Faculty	**Overall**^**b**^	Residents^b^	Faculty
*RB1. I understand challenges that residents face in receiving feedback from faculty*	**0.85**	0.78	1.00	**4.00 (0.57)**	3.94 (0.64)	4.14 (0.38)	**1.00**	1.00	1.00	**4.42 (0.50)**	4.44 (0.51)	4.33 (0.52)	**0.15**	0.22	0.00	**0.42 (0.15)** ***p* = 0.006372**	0.49 (0.20) *p* = 0.019444	0.19 (0.25) *p* = 0.459101
*RB2. I understand challenges that faculty face in providing feedback to residents*	**0.62**	0.44	1.00	**3.69 (0.62)**	3.44 (0.51)	4.29 (0.49)	**1.00**	1.00	1.00	**4.27 (0.45)**	4.25 (0.45)	4.33 (0.52)	**0.38**	0.56	0.00	**0.58 (0.15)** ***p* = 0.000345**	0.81 (0.16) *p* = 0.00003	0.05 (0.28) *p* = 0.867471
*RB3. I feel comfortable talking with both faculty members and residents about feedback*	**0.50**	0.33	0.86	**3.42 (0.95)**	3.06 (0.80)	4.29 (0.76)	**0.81**	0.75	0.83	**4.04 (0.66)**	3.94 (0.68)	4.33 (0.82)	**0.31**	0.42	-0.02	**0.62 (0.23)** ***p* = 0.00899**	0.88 (0.25) *p* = 0.001665	0.05 (0.44) *p* = 0.915035
*RB4. I am able to identify ways to address challenges related to giving and receiving feedback*	**0.42**	0.28	0.86	**3.35 (0.94)**	3.06 (0.87)	4.14 (0.69)	**0.92**	0.88	1.00	**4.23 (0.59)**	4.06 (0.57)	4.50 (0.55)	**0.50**	0.60	0.14	**0.88 (0.22)** ***p* = 0.00016**	1.01 (0.25) *p* = 0.000438	0.36 (0.34) *p* = 0.329648

^a^Aligning the five-category rating to a five-point Likert scale (i.e.: Strongly Disagree = 1, Disagree = 2, Neutral = 3, Agree = 4, Strongly Agree = 5)

^b^*p* < .05

Results of the post-workshop RB questionnaire items are displayed in Table 2. A large percentage of respondents selected “Strongly Agree” or “Agree” for items that assessed satisfaction with the workshop (RB5 through RB11). The mean score differences between resident and faculty responses for these seven items were not statistically significant.

**Table 2:** Results from rating-based post-workshop questionnaire items

	**Post-workshop questionnaire** **(*n* = 26 overall, including 16 residents, 6 faculty, and 4 unspecified)**					
**Rating-based (RB) questionnaire item**	Proportion of n selecting the Strongly Agree or Agree rating			Mean (SD) Likert scale responses^a^		
	**Overall**	Residents	Faculty	**Overall**	Residents	Faculty
*RB5. Overall, I was satisfied with this workshop*	**0.88**	0.88	1.00	**4.44 (0.65)**	4.38 (0.72)	4.50 (0.55)
*RB6. I learned new knowledge and skills from this workshop*	**1.00**	1.00	1.00	**4.23 (0.43)**	4.19 (0.40)	4.33 (0.52)
*RB7. I will be able to apply the knowledge and skills learned to improve my job performance*	**0.88**	0.81	1.00	**4.19 (0.63)**	4.00 (0.63)	4.50 (0.55)
*RB8. The scope of the workshop was appropriate for my professional needs*	**0.92**	0.94	0.83	**4.31 (0.62)**	4.25 (0.58)	4.50 (0.84)
*RB9. I would recommend this workshop to others*	**0.92**	0.88	1.00	**4.27 (0.60)**	4.19 (0.66)	4.50 (0.55)
*RB10. The learning activities and/or materials were effective in helping me learn the content*	**1.00**	1.00	1.00	**4.35 (0.49)**	4.31 (0.48)	4.50 (0.55)
*RB11. The content was presented in a fair and unbiased manner*	**1.00**	1.00	1.00	**4.54 (0.51)**	4.50 (0.52)	4.83 (0.41)

^a^Aligning the five-category rating to a five-point Likert scale (i.e.: Strongly Disagree = 1, Disagree = 2, Neutral = 3, Agree = 4, Strongly Agree = 5)

### Open-ended questionnaire responses

Analysis of responses to OE questions yielded twelve themes, falling into four broad categories – (i) desirable types of feedback, (ii) logistical and administrative circumstances, (iii) program norms and expectations, and (iv) relational and emotional considerations.

#### Desirable types of feedback

Respondents perceived (1) specific, (2) constructive, and (3) timely feedback to be desirable but challenging to both receive and provide. Resident respondents indicated, for example, that “often the feedback is not specific and seems like generalized feedback given to all residents,” and that “getting regular, consistent, and smaller bits of feedback” is rare. Faculty respondents similarly noted, for instance, that providing constructive feedback is difficult, especially to residents who are “underperforming or doing aberrant behavior.”

#### Logistical and administrative circumstances

Respondents perceived (4) finding time for feedback, (5) meeting formal evaluation requirements, and (6) improving based on feedback to be challenging. Resident responses included that the little time available for feedback is often spent completing required evaluation forms, and that it is not feasible for faculty to be “observing [a] resident in many interactions.” Faculty responses also reflected the limited opportunities for feedback when “most work [is] done independently,” and that residents often are not “having a chance to show improvement” following feedback.

#### Program norms and expectations

Respondents perceived the need for (7) development of effective feedback skills, (8) coordination of routine feedback sessions, and (9) residents to actively seek feedback. Resident respondents noted, for instance, that it is unclear “when to approach” faculty for feedback and how, and that “residents aren’t active in seeking feedback.” Faculty respondents similarly indicated, for example, that “not knowing how to give feedback” is a problem, and that “aligning feedback with learning objectives” is difficult, especially when there are no pre-established “expectations that feedback will happen.”

#### Relational and emotional considerations

Respondents perceived (10) rapport between resident and faculty, (11) fear of negative emotional impact, and (12) differences in interpersonal style to impact feedback practices. Resident responses included that there is “not enough positive rapport,” and that even “trusting positive feedback” can be challenging. Faculty responses included not wanting to “hurt resident feelings” or cause a “negative response from residents,” worrying that the intent of their feedback could potentially be misunderstood and taken personally.

There were some noteworthy changes from pre- to post-workshop in (i) residents’ view of what faculty find most challenging and (ii) faculty’s view of what residents find most challenging.

- Pre-workshop, 5 of 18 resident respondents indicated that they are uncertain of faculty’s challenges, and 4 of 18 respondents noted each of faculty’s difficulty finding time for feedback and fear of negative emotional impact. Post-workshop, no resident respondents mentioned being uncertain of faculty’s challenges. An increased number of respondents (7 of 16) pointed to faculty’s difficulty finding time for feedback, and 10 of 16 respondents commented on faculty’s fear of negative emotional impact, potentially indicating increased perspective taking by the residents of faculty thoughts about feedback.
- Pre-workshop, 4 of 7 faculty respondents perceived residents to fear negative emotional impact, and 3 of 7 respondents perceived residents to find receiving constructive feedback difficult. Post-workshop, notably less faculty respondents mentioned these points being what residents find most challenging (1 of 6 for each), 3 of 6 respondents pointed to residents finding timely feedback rare, and 4 of 6 respondents commented on residents being mainly concerned about rapport with faculty. These results potentially indicate increased perspective taking by the faculty of resident thoughts about feedback.

Open-ended (OE) questions OE3 through OE5 asked respondents for their thoughts on the workshop content and what future feedback workshops should focus on. Most respondents, both resident and faculty, found the workshop to be most useful in encouraging dialogue between residents and faculty and collaborative brainstorming. The workshop being held in person, its positive and inclusive tone, and provision of food were also liked by the respondents. Many respondents noted that the workshop would have been more useful if it had addressed specific feedback techniques/tools, included role play to practice using the techniques/tools, had more faculty participants, and allowed more time for brainstorming innovative improvements that can be made. Accordingly, respondents largely indicated wanting future workshops to be longer, be oriented towards practicing with specific techniques/tools, and with increased faculty participation.

## Discussion

This project is one of the first to examine a program aimed at enhancing understanding and appreciation of the different perspectives faculty and residents have of each other with regards to feedback. Findings indicated this approach increased the appreciation of the difficulties both faculty and residents experience giving and receiving feedback and generated strategies to overcome these difficulties. Both faculty and residents were satisfied with the programming, and had ideas for improvement – e.g., longer workshop duration, higher faculty participation, teaching of specific skills, increased role-playing.

Residents identified several factors as important to receiving feedback, including specificity of feedback, consistency of feedback, rapport with faculty, and protected time to receive feedback. Faculty identified several factors important to providing feedback, including protected time, skills training to provide effective feedback, and rapport with residents. Strategies to enhance provision of feedback included workshops on feedback, dissemination of specific successful strategies, and combined resident-faculty workshops.

Many of the challenges of giving and receiving feedback that the participants reported are well known. This work’s innovation is less about identifying new challenges experienced with feedback, and more about using a joint resident-faculty workshop to facilitate better understanding and appreciation of how each group views the feedback process. Importantly, as a pilot project, this work was focused on specifying the procedures involved in conducting and assessing the designed workshop, and not on testing a hypothesis that the workshop is effective. The strongest impact that new innovations such as this workshop can have on the field is by clearly describing and sharing their procedures for the innovative education initiative, so that the procedures can be replicated, adapted, and tested on larger scales through future work.

Although the project had strengths, there were also significant limitations in generalizing the results. This was a pilot project conducted as a quality improvement project. There was a small sample size and results may not generalize to a larger group of faculty and residents, or to other disciplines outside of psychiatry. Findings may also be subject to both (i) social desirability bias, as workshop participants may have completed the questionnaires with responses that they perceived to be desired by the authors and/or the training program, and (ii) researcher bias, as the authors may have interpreted the findings to align to what they expected to accomplish through this pilot project. An open invitation to attend the workshop was extended to multiple faculty members and residents, with no regard to ensuring the sample was representative of the faculty or resident populations. This limits the inferences that can be made about the themes identified by the qualitative data (this is unlike probability sampling for quantitative studies, which can lead to drawing statistical inferences about the prevalence of specified themes) [18, 19]. The authors were thus careful not to characterize the findings solely based on the frequency with which each theme is mentioned by participants (i.e., we did not attempt to quantify the qualitative results), beyond confirming the frequencies only to ensure that all data are accounted for [19, 20]. Relatedly, low faculty attendance must not be overlooked in interpreting the findings. In particular, faculty who chose to attend the workshop may have been those who already perceive feedback to be essential to training. To enable shared resident-faculty perspectives to reach additional faculty, future workshop organizers may consider working closely with training program leadership to better emphasize to faculty the program’s prioritization of enhanced feedback practices, and explicitly set attendance expectations for the workshop.

## Conclusions

Taken together, this is one of the first papers to examine a joint resident-faculty workshop to gain a better understanding of how each group perceives the process of giving and receiving feedback. Participating faculty and residents were satisfied with how the workshop led to their increased appreciation of each other’s difficulties in giving and receiving feedback, and they collaboratively brainstormed ideas for continuing to improve the residency training program’s feedback practices. Further work in this area is needed to identify common misperceptions about feedback and to develop programming that can help correct these. For instance, to determine generalizability of these findings, future studies may attempt to replicate this workshop at other institutions, or at cross-institution workshops (e.g., at professional development conferences such as the Association for Academic Psychiatry Annual Meeting). Additionally, follow-up (longer term) assessment of feedback workshop(s) is needed to understand their impact on changes in local feedback processes.

## Data Availability

The datasets used and/or analyzed during the current study are available from the corresponding author on reasonable request.

## Abbreviations

ACGME: Accreditation Council for Graduate Medical Education
HSS: Harvard South Shore Psychiatry Residency Training Program
OE: Open-ended
RB: Rating-based
VA: United States Department of Veterans Affairs
VABHS: VA Boston Healthcare System
